# Supplementary material for: Shifts in myeloarchitecture characterise adolescent development of cortical gradients
Source: eLife. 2019 Nov 14;8:e50482. doi: 10.7554/eLife.50482 (PMC6855802; doi:10.7554/eLife.50482)
Supplement: Supplementary file 1. [file elife-50482-supp1.docx]

#### Supplementary Table 1A: FDR corrected (Bonferroni) p-Values and Z-scores for age-related changes per level of laminar differentiation

|  | P-values | | | | Z-scores | | | |
| --- | --- | --- | --- | --- | --- | --- | --- | --- |
|  | mean | sd | skewness | kurtosis | mean | sd | skewness | kurtosis |
| heteromodal | 4.36E-02 | 1.27E-02 | 2.55E-08 | 2.69E-17 | 3.06 | 3.42 | 6.07 | 8.80 |
| idiotypic | 1.53E-07 | 1.00E+00 | 1.45E-04 | 1.76E-17 | -5.78 | -1.91 | -4.49 | -8.85 |
| paralimbic | 1.32E-02 | 4.39E-07 | 7.21E-12 | 1.23E-05 | -3.40 | -5.60 | -7.27 | -4.99 |
| unimodal | 1.90E-04 | 2.85E-01 | 2.43E-02 | 8.38E-01 | 4.43 | 2.45 | 3.24 | 2.03 |

#### Supplementary Table 1B: FDR corrected (Bonferroni) P-Values and Z-scores for age-related changes per Von Economo structural type

|  | P-values | | | | Z-scores | | | |
| --- | --- | --- | --- | --- | --- | --- | --- | --- |
|  | mean | sd | skewness | kurtosis | mean | sd | skewness | kurtosis |
| association1 | 6.86E-01 | 3.31E-12 | 5.20E-13 | 5.97E-11 | 2.30 | 7.44 | 7.68 | 7.04 |
| association2 | 1.43E-04 | 1.91E-01 | 1.63E-02 | 1.00E+00 | 4.59 | 2.75 | 3.48 | 2.12 |
| insula | 1.00E+00 | 6.97E-01 | 1.00E+00 | 1.00E+00 | -0.25 | -2.29 | -1.42 | -0.73 |
| limbic | 4.14E-04 | 1.39E-04 | 5.98E-04 | 1.00E+00 | -4.36 | -4.59 | -4.28 | -1.95 |
| motor | 1.27E-02 | 1.00E+00 | 7.70E-03 | 1.04E-06 | -3.54 | 0.37 | 3.67 | -5.53 |
| primary sensory | 1.90E-02 | 1.00E+00 | 2.67E-06 | 1.62E-04 | -3.43 | 0.51 | -5.36 | -4.56 |
| secondary sensory | 1.00E+00 | 1.99E-13 | 8.57E-11 | 5.59E-01 | 0.81 | -7.80 | -6.99 | -2.38 |
